# Vitamin B6 Is Under a Tight Balance During Disease Development by *Rhizoctonia solani* on Different Cultivars of Potato and on *Arabidopsis thaliana* Mutants

**DOI:** 10.3389/fpls.2020.00875

**Published:** 2020-06-24

**Authors:** Jamil Samsatly, Stéphane Bayen, Suha H. Jabaji

**Affiliations:** ^1^Department of Plant Science, McGill University, Sainte-Anne-de-Bellevue, QC, Canada; ^2^Department of Food Science and Agricultural Chemistry, McGill University, Sainte-Anne-de-Bellevue, QC, Canada

**Keywords:** vitamin B6, *de novo* pathway, salvage pathway, *Rhizoctonia solani*, oxidative stress, biotic stress, *PDX* genes, antioxidant genes

## Abstract

Vitamin B6 is well recognized as an essential antioxidant and plays a role in stress responses. Co-expression of plant and pathogen-derived vitamin B6 genes is critical during disease development of *R. solani*. However, little is known about the functionality of vitamin B6 vitamers during plant-*R. solani* interactions and their involvement in disease tolerance. Here, we explored the possible involvement of vitamin B6 during disease progression of potato cultivars of different susceptibility levels to *R. solani*. A distinct pattern of gene expression, pyridoxine (PN) concentration, and fungal biomass was found in the susceptible cv. Russet Burbank and tolerant cv. Chieftain. Accumulation of reactive oxygen species (ROS) in *R. solani* mycelia or plant tissues applying non-fluorescence or fluorescence methods was related to up-regulation in the vitamin B6 pathway and is indicative of oxidative stress. Russet Burbank was susceptible to *R. solani*, which was linked to reduced amounts of VB6 content. Prior to infection, constitutive PN levels were significantly higher in Russet Burbank by 1.6-fold compared to Chieftain. Upon infection with *R. solani*, PN levels in infected tissues increased more in Chieftain (1.7-fold) compared to Russet Burbank (1.4-fold). *R. solani* AG3 infection of potato sprouts in both cultivars significantly activates the fungal and plant vitamin B6 and glutathione-*S*-transferase (GST) genes in a tissue-specific response. Significant fold increases of transcript abundance of the fungal genes ranged from a minimum of 3.60 (*RsolSG3GST*) to a maximum of 13.91 (*RsolAG3PDX2*) in the surrounding necrotic lesion tissues (zone 1). On the other hand, PCA showed that the top plant genes *STGST* and *STPDX1.1* were linked to both tissues of necrotic lesions (zone 2) and their surrounding areas of necrotic lesions. Functional characterization of Arabidopsis *pdx1.3* mutants challenged with *R. solani* provided evidence into the role of the vitamin B6 pathway in the maintenance of plant tolerance during disease progression. Overall, we demonstrate that the production of vitamin VB6 is under tight control and is an essential determinant of disease development during the interaction of *R. solani* with potato cultivars.

## Introduction

Rhizoctonia disease of potato or blackscurf, caused by *Rhizoctonia solani* Kuhn (teliomorph T*hanatephorus cucumeris* Frank Donk), occurs in most potato producing areas worldwide. Isolates of *R. solani* anastomosis group AG3 are recognized as the etiological agents for this disease. Disease symptoms manifest on potato sprouts, stolons, and roots, causing sprout nipping and cankers, which are reddish to brown lesions on stolons and roots (Gutierrez et al., 1997). Severe damage at this stage leads to uneven crop stand, and reduced yield (Hide and Horrocks, 1994; Hide et al., 1996). Tubers are also affected with the appearance of black sclerotia (resting vegetative bodies of the fungus). To date, no variety has immunity to the sprout nipping and stem lesion phase, and breeding for resistance does not control the pathogen. However, some varieties show varying degrees of resistance to the formation of sclerotia on tubers.

Like many other necrotrophic pathogens, *R. solani* causes extensive necrosis and tissue maceration (Laluk and Mengiste, 2010). The shared elements in early disease development of *R. solani* isolates are the intimate association of fungal hyphae with the epidermis of the host forming infection cushion or aggregates, tissue penetration, and maceration by cell wall degrading enzymes and other enzymes which eventually leads to tissue browning associated with high levels of reactive oxygen species (ROS) and tissue death (Keijer, 1996; Sneh et al., 2013). ROS molecules are highly reactive and toxic to biological molecules, and are not only essential regulators of plant growth, but are involved in limiting pathogen spread, induction of cell death and signal transduction in host-plant interactions (Torres, 2010; Barna et al., 2012). There is also increasing evidence that fungi also produce ROS during pathogenic interactions (Daub and Ehrenshaft, 2000; Samsatly et al., 2018). Therefore, the regulation of ROS in fungal cells and tolerance to external ROS produced by the host plant represent a balanced control and detoxification by both partners which can govern the fate of disease progression of necrotrophic pathogens (Heller and Tudzynski, 2011). To fine-tune and maintain this balance, plant and fungal cells possess a complex battery of protective mechanisms such as oxalic acid or the NADPH oxidase RBohD (Torres et al., 2005; Kadota et al., 2015), or ROS-quenching molecules including vitamin B6 (VB6) and several antioxidation enzymes including superoxide dismutase (SOD), catalase (CAT), ascorbate peroxidase (APX), glutathione -S-transferase (GST), and glutathione reductase (Das and Roychoudhury, 2014; Samsatly et al., 2015; Zhang et al., 2015; Girard et al., 2017). In the case of *R. solani*, the initiation of basal resistance in plants to *R. solani* in different pathosystems is closely linked to ROS-scavenging mechanisms, oxylipins production, cell wall-bound phenolic compounds, and the build-up of metabolites related to vitamin B6 biosynthetic pathway (Taheri and Tarighi, 2011; Nikraftar et al., 2013; Aliferis et al., 2014; Foley et al., 2016; Samsatly et al., 2018).

Vitamin B6 plays a critical role in all living organisms and is recognized as an important cofactor required for many enzymatic reactions (Hellmann and Mooney, 2010). Vitamin B6 is a collection of six vitamers: pyridoxine (PN), pyridoxal (PL), pyridoxamine (PM), and their phosphorylated derivatives (Fitzpatrick et al., 2012; Vanderschuren et al., 2013). In plants and fungi, the *de novo* biosynthesis of VB6 vitamers (the so-called DXP-independent pathway) requires two pyridoxal protein families (PDX1 and PDX2) that are highly conserved. PDX1 and PDX2 proteins contain the glutamine amidotransferase where PDX1 functions as the synthase and PDX2 constitutes the glutaminase domain (Raschle et al., 2005; Fitzpatrick et al., 2007). Another important component of the VB6 biosynthetic machinery is the salvage pathway, which is responsible for the interconversion between the different forms of VB6 vitamers (González-Guerrero et al., 2007; Herrero et al., 2011; Rueschhoff et al., 2012). It includes pyridoxal reductase (PLR), a downstream enzyme in the VB6 biosynthesis pathway, which converts PL into PN (Morita et al., 2004; Herrero et al., 2011).

In the last decade, the regulatory function of key genes involved in the VB6 biosynthetic pathways was characterized in the model plant Arabidopsis, recognizing the importance of VB6 in plant development and response to stress (Hellmann and Mooney, 2010). There are three *PDX1* homologs in Arabidopsis, two (*PDX1.1* and *PDX1.3*) of which are catalytically active, and they encode PDX (Titiz et al., 2006; Tambasco-Studart et al., 2007). PDX1.3 is required for disease resistance to bacterial and fungal diseases in Arabidopsis (Moccand et al., 2014; Zhang et al., 2014, 2015). In contrast to Arabidopsis, potato (*Solanum tuberosum* L.) has only two *PDX1* homologs (*PDX1.1* and *PDX1.2*), and only *PDX1.1* is catalytically active (Mooney et al., 2013). Several studies had shown that VB6 is a potent antioxidant with quenching ROS ability that matches that of tocopherols or ascorbic acid. This novel role of VB6 as ROS scavenger and its ability to increase resistance against biotic and abiotic types of stress has been demonstrated in microbe-microbe (Chamoun and Jabaji, 2011; Gkarmiri et al., 2015) and in plant-microbe interactions (Ehrenshaft et al., 1999; Benabdellah et al., 2009; Mooney et al., 2009; Vanderschuren et al., 2013). During plant-microbe interactions, the plant-derived VB6 genes, involved in the *de novo* biosynthetic pathway, were upregulated in response to various stresses (Denslow et al., 2005; Bagri et al., 2018; Chandrasekaran and Chun, 2018; Samsatly et al., 2018). These results imply that VB6 vitamers might contribute to disease resistance in modulating plant defense responses against different types of pathogens. Zhang et al. (2014) provided direct evidence supporting this notion by examining Arabidopsis mutant ecotypes that are defective in the *de novo*, and the salvage pathways of VB6 biosynthesis. Mutations in the Arabidopsis *PDX1.2* and *PDX1.3* genes involved in the *de novo* pathway increased disease levels caused by *Botrytis cinerea* and decreased the content level of VB6 (Zhang et al., 2014, 2015). Despite these studies on the regulation and co-expression of VB6 genes in the host and the pathogen of the *de novo* and salvage biosynthetic pathways during host–pathogen interactions remain limited to the best of our knowledge to only one study (Samsatly et al., 2018).

In a preceding research, we provided evidence that genes of vitamin B6 biosynthetic machinery are implicated as antioxidants in response to oxidative stress formed during potato-*R. solani* interaction and that VB6 genes are co-expressed in both the potato and *R. solani* during their interaction. We also provided evidence that the differential transcription of genes in infected necrotic tissues and their surrounding areas is spatially regulated (Samsatly et al., 2018). These results indicate that VB6 antioxidant genes are critical during disease development from the host’s viewpoint, and are crucial for detoxification and pathogenicity from the pathogen’s perspective. Therefore, developing new strategies to combat yield losses in potato requires a thorough understanding of the antioxidant genes and gene regulatory networks underlying the plant defense response, especially in existing tolerant breeding potato cultivars.

In this study, we expanded the research to first, test whether potato genotypes containing variable amounts of VB6 (Mooney et al., 2013) would exhibit distinct degrees of tolerance to *R. solani* AG3, and that this tolerance is tightly linked with the differential expression of genes of VB6 *de novo* and salvage biosynthetic pathways. Second, we used a LC-MS-based method to report on the content of the VB6 vitamer, pyridoxine (PN) in infected potato cultivars and to assess *R. solani-*induced expression of VB6 biosynthetic genes. Third, we examined phenotypes of Arabidopsis mutants with defects in the *de novo* pathway of VB6 biosynthesis against infection by *R. solani*, compared their VB6 content, and providing evidence that mutations in *PDX1.3* led to increased disease, suggesting the role of VB6 in plant resistance.

## Materials and Methods

### Biological Material and Inoculation

#### *Rhizoctonia solani* Inoculum Preparation

Cultures of *R. solani* AG3 isolate Rs114 (ATCC 10183), a pathogen of potato, and *R. solani* AG4 (subgroup HG-I) isolate A76, a pathogen of Arabidopsis was provided by M. Cubeta, North Carolina University, United States. Strains were retrieved from 10% glycerol stock stored at −80°C on potato dextrose agar (PDA; Difco Laboratories) and grown for 3 days at 24°C in the dark and used as a source of inoculum for plant infection. Strips of PDA (2 cm × 8 cm) containing mycelia growth *R. solani* served as the inoculum. For RNA and DNA extraction, cultures of *R. solani* strains of AG3 and AG4 were grown on PDA overlaid with cellophane membrane following the method of Samsatly et al. (2018).

#### *Solanum tuberosum* L. Growth Conditions

Certified potato tubers with different susceptibility levels to *R. solani* were used in this study. *Solanum tuberosum* cv. Russet Burbank, susceptible to *R. solani* (Thangavel et al., 2014) was provided by D.J. Donnelly (McGill University), and *S. tuberosum* cv. Chieftain, a tolerant to *R. solani* (Dowley, 1972) was obtained from the Northern Horticultural Research/SPUD Unit, New Liskeard Agricultural Research Station, University of Guelph (Canada). For sprout induction, uniform-sized tubers, previously kept at 4°C in the dark, were sprouted for 12 days under controlled conditions following the method of Samsatly et al. (2018). Sprout tips of 3 cm in length were sprayed with 1.5 mL solution of CaNO_3_ (0.5 M) to prevent browning. Inoculation of sprouts with *R. solani* was performed when sprouts were 8 cm in length.

#### Sprout Inoculation and Tissue Collection

To induce *R. solani* infections on potato sprouts, excised sprouts (8 cm in length) were sandwiched with two PDA strips (2 cm × 8 cm) of a 3-day-old *R. solani* AG3 Rs114 culture and placed in sterile Pyrex trays (40 cm × 26 cm) lined with wet sterile Whatman No. 1 paper as fully described in Samsatly et al. (2018). Mock-inoculated control potato sprouts were sandwiched with sterile PDA strips. There were 3 biological replicates per treatment and each treatment replicate consisted of 4 excised potato sprouts.

**FIGURE 1:**
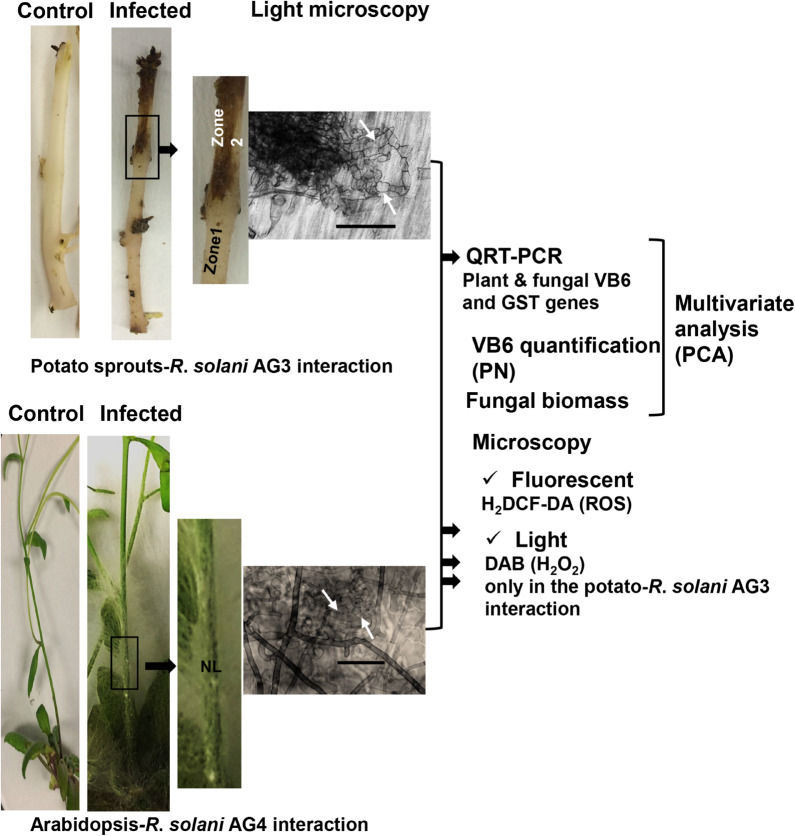
Flow chart of the experimental set-up for disease development by *R. solani* AG3 on potato sprouts and by *R*. *solani* AG4 on Arabidposis. Sprouts and Arabidopsis lower stems were sandwiched between two PDA strips of a 3-day-old *R*. *solani* AG3 or AG4 cultures, respectively. Sprouts or Arabidopsis lower stems sandwiched with sterile PDA strips served as controls. Samples were taken at 120 HPI (Hour post-inoculation) (Potato-*R. solani* AG3 interaction) from adjacent surrounding areas (Zone 1) and necrotic lesions (Zone 2), and 72 HPI (Arabidopsis-*R. solani* AG4 interaction) from infected lower stems of Arabidopsis. Corresponding segments were taken from controls. Typical initials of infection cushions with mycelial mass aggregates are clearly visible under the light microscope (white arrows). Transcriptional abundance of antioxidant genes, PN concentrations, and fungal biomass from both the plant and pathogen was analyzed in zones1 and 2 (Potato-*R. solani* AG3 interaction) and lower stem areas (Arabidopsis-*R. solani* AG4 interaction) followed by multivariate analysis (PCA). The two interactions were examined for ROS accumulation using fluorescent (H_2_DCF-DA) and DAB (only the potato interaction) staining methods in control and infected sprouts or Arabidopsis lower stems. Zone1: tissue surrounding necrotic lesion. Zone 2, necrotic lesion; NL, necrotic lesion. Bar = 50 μm.

Pyrex trays were covered with transparent plastic film, and placed in a growth chamber under photosynthetically inactive black light (365 nm) at 24°C. PDA strips were removed 120 h post-inoculation (HPI) of potato sprouts to reveal the infection areas with visible necrotic lesions on the sprouts. This time point was optimal to secure the beginning of infection cushions and mycelial aggregates that were stereoscopically examined (Figure 1). Two distinct zones were sampled from potato stolons. Zone 1 consisted of a one-cm area bordering the lesions and zone 2 contained the necrotic lesions with infection structures of *R. solani* (Figure 1). Similar areas and amounts of tissues were excised from mock-inoculated controls (Figure 1). Tissues were flash-frozen with liquid nitrogen and stored at −80°C for downstream applications.

#### Optical and Fluorescence Microscopy

The accumulation of cellular and extracellular ROS, primarily peroxide (H_2_O_2_) during disease progression in tissues of infected potato and mock-inoculated was visualized following the method of Samsatly et al. (2018). Briefly, ROS detection in tissue samples from potato were incubated with 10 μM of the fluorescent probe, 2′,7′-dichlorodihydrofluorescein diacetate (H_2_DCF-DA) in H_2_O for 30 min, followed by washing with prewarmed H_2_O to remove the non-internalized probe. For the detection of *in situ* hydrogen peroxide (H_2_O_2__)_ accumulation, thin shavings of potato tissues of infected and mock-inoculated control were stained with 3,3’-diaminobenzidine (DAB) followed by clearing with 15.1M of chloral hydrate solution. Samples were examined under Zeiss SteREO Discovery.V20 microscope (Carl Zeiss Canada Ltd., Toronto, ON, Canada). Fluorescence detection in plant tissues was read at an excitation wavelength of 470 nm using a GFP filter. Bright field microscopy was performed to monitor disease development and capture fungal infection cushions, and images were digitally documented with the Moticam 2300 digital camera (GENEQ Inc., Montreal, QC, Canada) for light microscopy.

#### Quantification of Fungal Load in Infected Potato Sprouts

The quantification of fungal load by quantitative QPCR is a reliable method to measure fungal biomass during infection especially in pathogens, such as *R. solani* that do not produce conidia (Baumgartner et al., 2010), and also to assess fungal load of necrotrophic pathogens in Arabidopsis (Gachon and Saindrenan, 2004). We have estimated *R. solani* DNA abundance of the glyceraldehyde-3-phosphatedehydrogenase gene in infected potato tissues of both cultivars by quantitative QPCR using the AG-3 specific primer pair set G3PDH-F/R (Table 1). The putative amplified products were purified using the QIAquick PCR Purification Kit (Qiagen), and sub-cloned in pDrive cloning vector (Qiagen). Absolute quantification of genomic gene copies was established from standard curves using six consecutive 10-fold dilutions of genomic DNA for *R. solani* AG3. Initial copies were calculated using the genomic size following the method of King et al. (2015). Total genomic DNA was isolated from 100 mg of mock-inoculated control plant tissues and infected tissues (necrotic lesions) of potato and Arabidopsis using the DNeasy Plant Mini Kit^TM^ (Qiagen, Toronto, ON, Canada). The concentration and purity of DNA were assessed by spectrophotometry with ND1000 (NanoDrop, Wilmington, Delaware). All Q-PCR reactions were carried on using Stratagene Mx3000 (Stratagene, Cedar Creek, United States).

**TABLE 1:** List of primers used in this study.

Target organism	Primer	Sequence (5′→3′)	Annealing temperature (°C)	Amplicon size (bp)	References
	***PDX1* (Pyridoxine biosynthesis gene)**				
Arabidopsis	*At^*a*^- PDX1.1-F*	CGAAGGAGCCATGACGGAGA	58	196	At2G38230
	*At-PDX1.1-R*	AGCAACACCGCCTTGAGCTCGA			
	*At-PDX1.2-F*	TGTTACTCTCTACAGCGGCAC	54	130	At3G16050
	*At-PDX1.2-R*	CTTAGCTTGGTTAACCGAGGAA			
	*At-PDX1.3-F*	TAACGGTGCGATAACGGAGG	53	231	AT5G01410
	*At-PDX1.3-R*	TGTTTGATTTCTTTAATCATTTG			
Potato	*ST*^*b*^-*PDX1.1-qF*	CTGTGACTATTCCTGTAAT	55	83	Mooney et al., 2013
	*ST*-*PDX1.1-qR*	GTAATCTACTCCGATAGC			
	*ST*-*PDX1.2-qF*	TGCTCTAATCCTTACAAG	55	162	
	*ST-PDX1.2-qR*	GTAGGTCTCATCACTAAC			
*R. solani* AG3	*5′GSP1-(PDX1)*	TGACGAACAGCCTCGACAACATTTCC	55	219	Samsatly et al., 2015
	*3′GSP2-(PDX1)*	TCCGTTTGTCTGTGGGGCTACATCTCTC			
*R. solani* AG4	*Rsol^*c*^AG4-PDX1- F*	ATGATCAAAGAGATCGTGGAC	53	243	AVOZ01001318.1
	*RsolAG4-PDX1-R*	GATCATTGCTGCACCTTCGGA			
	***PDX*2 (Pyridoxine biosynthesis gene)**				
Arabidopsis	*At-PDX2-F*	ACTAACATCTCAAGAAGGTGG	53	145	AT5G60540
	*At-PDX2-R*	TTGAATTTGTACGGTGGAGCTT			
Potato	*ST-PDX2-qF*	ATTCCAATCCTGCTATTC	55	185	Mooney et al., 2013
	*ST-PDX2-qR*	CACAATATCAGAAGTTCCT			
*R. solani AG3*	*3′GSP1-(PDX2)*	AATCTTGCTTGCCTCTGGTGGTGTTG	55	225	Samsatly et al., 2015
	*5′GSP1-(PDX2)*	ATCCCATTAAATGGTCGGTCCTCATCA			
*R. solani AG4*	*RsolAG8-PDX2-F*	ATTATATCACGCGTGACACC	53	207	JATN01000314.1
	*RsolAG8-PDX2-R*	GGCGCCTTCAACACCACCAGA			
	**PLR (pyridoxal reductase)**				
Arabidopsis	*At -PLR-F*	TGGGTCCTTTAAGTGTTTCT	53	170	AT5G53580
	*At -PLR-R*	TTTGGCCATTAAGCCTACCAGTG			
Potato	*ST-PLR-F*	GCCGCCCAAACCCGAACCCGA	58	118	XM_006358747.2
	*ST-PLR-R*	AAGGAGGATCTCGTTAGTGT			
*R. solani* AG3	*PLR AKR8 F*	GAAAGCCTCCTCTTGGAATCT	58	300	Chamoun and Jabaji, 2011
	*PLR AKR8 R*	GGGTAAGATTGGATCGATTGGG			
*R. solani* AG4	*RsolAG1-IA-PLR-F*	AAGGGGCACTAACCGGAAAGC	58	194	AFRT01000983.1
	*RsolAG1-IA-PLR-R*	ATGTTGCCCGAGAGAGGAGAC			
	**Glutathione S-Transferases**				
Arabidopsis	*At -GST^d^-F*	TGGCTCTCAAGCTTAAAGGCG	54	213	AT2G29450
	*At -GST-R*	TGTCATATGGACTTTGCGGTA			
Potato	*ST-GST-F*	TCCTTTTAGCCATAGAGTTGA	51	200	POTPR1A
	*ST-GST-R*	CTTCAAATGCCTCATCAATGC			
*R. solani* AG3	*RsolAG3-GST-F*	AGAAGACGAGGCAAATGCGA	57	256	Samsatly et al., 2015
	*RsolAG3-GST-R*	ATCTCTTCAACCGCCTTCCAGT			
*R. solani* AG4	*RsolAG1-IA-GST-F*	TACAAACGCATACCTTATCGC	53	217	ELU40094.1
	*RsolAG1-IA-GST -R*	TTGGAGCCGGGTAAGTTTTG			
	**Reference genes**				
Arabidopsis	*PP2AA3-^*e*^ _F*	TAACGTGGCCAAAATGATGC	53	60	Libault et al., 2008
	*PP2AA3_R*	GTTCTCCACAACCGCTTGGT			
	*At-TIP41^*f*^-F*	GTGAAAACTGTTGGAGAGAAGCAA	55	60	Nardeli et al., 2017
	*At-TIP41-R*	TCAACTGGATACCCTTTCGCA			
Potato	*ST-Actin7-qF*	GGCTATGTATGTTGCTAT	55	186	Mooney et al., 2013
	*ST-Actin7-qR*	ATCTTCATCAGGTTATCAG			
*R. solani* AG3	*G3PDH^*g*^ -F*	GGTATTATTGGATACACTGA	55	129	Chamoun and Jabaji, 2011
	*G3PDH -R*	TTAAGCCTCAGCGTCTTTCT			
*R. solani* AG4	*GM-RS-4^*h*^*	CGGTTCATCTGCATTTACCTT	55	88	Johanson et al., 1998
	*GM-RS3-R*	AGTGTTATGCTTGGTTCCACT			
	**Genotyping primers**				
Arabidopsis	*SALK 086418-LP SALK 086418-RP*	AGCGAACCTCTCAACCTTCTC TGTTTTAATCTTGACCGTCCG	60	534-834	http://signal.salk.edu/tdnaprimers.2.html
	SALK LB-1.3	ATTTTGCCGATTTCGGAAC	60		

*Primer names were preceded by the plant or fungus abbreviation: ST, Solanum tuberosum, AT, Arabidopsis thaliana, or Rsol, R. solani. QRT-PCR conditions were optimized for each primer set, and products were confirmed by sequencing. Internal reference genes for each plant type and fungal strain (Table 1) were used for normalization of relative transcript abundance levels. ^*a*^Arabidopsis thaliana. ^*b*^Solanum tuberosum. ^*c*^Rhizoctonia solani. ^*d*^Glutathione S-Transferase. ^*e*^Protein phosphatase 2A subunit A3. ^*f*^TIP41-like family protein. ^*g*^G3PDH: Glyceraldehyde-3-phosphate dehydrogenase. ^*h*^ITS1 of 5.8S rRNA.*

Since fungal load is not a direct measure of disease progression, we digitally analyzed disease progression on sprout sections of both potato cultivars using the software ImageJ (Rasband, W.S., ImageJ; U.S. National Institutes of Health, Bethesda, MD, United States) (1997–2012)^1^. The disease index was calculated as percent area of tissue infected by *R. solani* AG3 compared to the total area of tissue for Burbank and Chieftain cultivars using software ImageJ (Abd-El-Haliem, 2012; Stewart et al., 2016).

#### Vitamin B6 Analysis by LC-MS

To determine whether the level of vitamin B6 was affected in infected tissues as compared to mock-inoculated controls, the vitamer pyridoxine (PN) was extracted from lyophilized inoculated and mock-inoculated control tissues of potato cultivars following the modified method of Denslow et al. (2005). Briefly, 0.1 g of tissues were ground in liquid nitrogen, mixed with 30 mL 0.44 N HCl, and autoclaved at 121°C for 2 h. pH was brought to 4.8 using sodium acetate, and each sample was de-glycosylated using fresh B-glucosidase (5 mg in 1 mL H_2_O) and de-phosphorylated with fresh acid phosphatase (75 mg in 2 mL H_2_O). Samples were added to flasks (50 mL capacity) and incubated overnight in a 37°C incubator shaker at 70 rpm. The mixture in each flask was brought to 50 mL with deionized H_2_O and filtered through 0.22 μm filters (Millex-FG, Millipore, MA, United States). Samples were stored at −20°C and all later steps of vitamin B6 analysis were done in dark conditions. Surrogate analog, 400 ng of pyridoxine-(methyl-d3) (Sigma-Aldrich, Oakville, Canada) (Table 2), was spiked before extraction in all samples. Procedural blanks were prepared in the same manner. Four samples were spiked with 400 ng of PN (Sigma-Aldrich) to estimate the recovery rates of the method. Matrix effects were assessed by comparing the signal obtained from spiked extracts with PN standard and with the equivalent signal obtained from pure solvent standards. Unless otherwise stated, VB6 concentrations refer to the amounts of the vitamer PN.

**TABLE 2:** Vitamin B6 vitamers used in the study and their description.

Vitamer	Mw g/mol	[M + H]^+^ *m/z*	Empirical Formula	Retention time (minute)
Pyridoxine-(methyl-d3)	172.1	173.10	C_8_D_3_H_8_NO_3_	2.33
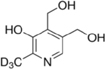				
Pyridoxine (PN)	169.1	170.08	C_8_H_11_NO_3_	2.33
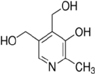				

High-performance liquid chromatography coupled to quadrupole time-of-flight tandem mass spectrometry (HPLC-QTOF-MS) analysis was performed using an Agilent 1290 Infinity LC System coupled to a 6540 Accurate-Mass QTOF System operated in the positive ionization mode (Agilent Technologies, Santa Clara, CA, United States). The separation was performed with a Poroshell EC-C18 Column (3.0 × 100 mm, 2.7 μm, Agilent Technologies) fitted with an Infinity Lab Poroshell 120 pre-column (3.0 × 5 mm, 2.7 μm) in a thermo-stated column compartment set at 20°C. HPLC grade solvents (water, methanol), as well as LC/MS grade of formic acid were all purchased from Fisher Chemical (Pittsburgh, PA, United States) (Supplementary Figure S1). The mobile phase consisted of water (A) and methanol (B), both containing 0.1% of formic acid. The HPLC gradient was as follows: 1–6 min (100% A), 6–10 min (75% A and 25% B), 10–12 (100% B) min, 12–12.01 min (100% B), 12.01–14 min (100% B). The flow rate was 0.5 mL.min^–1^ with an injection volume of 2.00 μL. MS scans (*m/z* 50–500) were completed at a scan rate of 4 spectra/s. Prior to analysis, the QTOF was tuned (mass accuracy < 1 ppm), and mass accuracy was maintained throughout the batch using the continuous infusion of a reference mass mix. Compound identification was based on mass spectra and retention times of pure VB6 analytical standards (Table 2). Target analytes were quantified using a 6-points calibration range (10–400 ng.mL^–1^) based on the extracted chromatogram for their specific protonated molecular ion [M + H]^+^. Concentrations were calculated from the relative response versus the mass-labeled PN analog.

#### RNA Extraction, and Quantitative RT-PCR

Total RNA was extracted from flash-frozen pulverized tissues (100 mg) using TRIZOL (Generay Biotech, Shanghai, China) following the manufacturer’s instructions. RNA (500 ng) was reverse-transcribed with the Quantitect Reverse transcriptase kit^TM^ (Qiagen) according to the manufacturer’s protocols. QRT-PCR analyses were conducted on fungal and plant target antioxidant genes (Table 1). Primers for fungal VB6 and *GST* encoding genes were designed as previously described (Samsatly et al., 2018). Internal reference genes and potato VB6 and *GST* encoding genes were synthesized based on published sequenced primers (Table 1). QRT-PCR conditions were optimized for each primer set (Table 1), and putative products were confirmed by sequencing. Reverse transcription PCR assays were performed on three biological replicates and two technical replicates. QRT-PCR assays were conducted on fungal and plant target antioxidant genes encoding *GST*, the *de novo* VB6 biosynthesis genes; a synthase (*PDX1*) and a *glutaminase* (*PDX2*), and the VB6 salvage pathway encoding gene, *Pyridoxal Reductase* (*PLR*) and appropriate internal reference genes (Table 1) using Stratagene Mx3000 (Stratagene, Cedar Creek, United States). PCR assay conditions were performed using suitable annealing temperature for each primer pair (Table 1) with the following conditions: Each 20 μL reaction contained 1X SsoAdvanced Universal SYBR Green Supermix (Bio-Rad Laboratories Ltd.), 0.16 μM each primer, and 500 ng cDNA. The thermocycling profile used an initial denaturation at 98°C for 2 min, followed by 35 or 40 cycles of denaturation at 98°C for 15 s, annealing for 30 s at the appropriate primer temperature and extension at 72°C for 30 s, followed by a dissociation curve analysis. At every run, routine negative and positive controls were performed. The relative transcript abundance levels of the plant and fungal-derived genes were normalized against their respective reference genes according to the method reported by Zhoa and Fernald (2005).

#### Arabidopsis Pathogenicity Experiments

Seeds of *Arabidopsis thaliana* wild type, ecotype Col-0 and *pdx1.3* mutants (At5g01410- SALK 086418C) were obtained from Arabidopsis Biological Resource Center (Ohio State University, Columbus; (Alonso et al., 2003; Titiz et al., 2006). PCR-based genotyping was carried out for SALK 086418C lines to screen for homozygous plants. Gene-specific primers SALK 086418-LP/RP and T-DNA primers SALK LB-1.3 (Table 1) were included following the T-DNA Express Web site^2^. Plants homozygous for PDX1.3 mutation were used for downstream applications.

Seeds were surface sterilized by immersing them in 70% ethanol for 45 s, followed by 1.3% bleach for 3 min and rinsed five times with sterile water. Sterile seeds were stratified at 4°C for 5 days in the dark prior to planting. Seeds were sown in Square Pots 10 × 10 × 11 cm 0.69 L containing AgroMix G10 (Fafard Ltd.) and sand (1:1, v/v) with one seed per pot and placed in a growth cabinet with a 16/8 h light/dark photoperiod and 100 μmol/ms of light at 22°C/19°C, respectively, with 60% relative humidity. Eight weeks-old seedlings were used for disease assays.

Arabidopsis seedlings of wild type and mutant ecotypes were carefully removed from the potting mix, and the roots were rinsed with distilled water to remove soil particles. Ten seedlings of Arabidopsis wild type ecotype Col-0 and *pdx1.3* mutants (At5g01410, SALK 086418C) were placed in sterile Pyrex trays as described above and sandwiched with two strips of PDA strips (0.5 cm × 8 cm) of a 3-day-old *R. solani* AG4 isolate A76 culture covering the crown, lower stem and part of the roots. Mock-inoculated controls were sandwiched with sterile PDA strips. There were three biological replicates per treatment and each treatment replicate consisted of 10 Arabidopsis seedlings. Progression of infection was determined at 72 HPI. One zone was sampled which consists of necrotic lesions developing at the crown area and lower stem parts. Similar areas and amounts of tissues were excised from mock-inoculated controls.

RNA isolation, cDNA synthesis, and qPCR reactions were carried out as described above for potato. Primer sets were designed for Arabidopsis based on sequences from NCBI, and were checked for specificity to amplify only their target gene (Table 1). Specific primers used for the amplification of VB6, *GST* encoding genes of Arabidopsis and of *R. solani* AG4 were designed as previously described (Samsatly et al., 2018). Internal reference genes for Arabidopsis and *R. solani* AG4 (Table 1) were used for normalization of relative transcript abundance levels.

The fungal load of *R. solani* AG4 was estimated in infected tissues of Arabidopsis wild type and *pdx1.3* mutant line, by amplifying the gDNA using the primer pair set RsolAG4-PDX1F/R in Q-PCR assays (Table 1). PCR assays were performed as described above for potato. Optical and fluorescence microscopy to detect ROS accumulation, primarily superoxide (O^–^_2_) and hydrogen peroxide (H_2_O_2_) during disease progression in the wild type and mutant lines of Arabidopsis infected or not with *R. solani* AG4 was visualized by H_2_DCF-DA and staining method following the protocol described above.

#### Statistical Analyses

Two-way analysis of variance (ANOVA) and the least significant differences (LSD) at *P* < 0.05 were used to compare the values of the relative transcript abundance, VB6 concentrations and fungal biomass between the treatments and the controls using the SPSS statistical package v. 22.0 (IBM Corp., Armonk, NY, United States). Transcript changes were considered statistically and biologically significant if *P* < 0.05 and fold changes were >+ 1.5 or >-1.5.

In order to understand the simultaneous relationships among the relative abundance of host and fungal-derived antioxidant genes, VB6 concentrations and fungal biomass, and to predict the effect and impact of a change in one variable will have on other variables, a multivariate analysis of the data was performed on potato-*R. solani* and Arabidopsis-*R. solani* interactions (Figure 1) using the SIMAC-P + v12.0 (Umetrics, MKS Instruments Inc., Andover, MA, United States). The data matrix for potato-*R. solani* comprised of biological replicates of control fungal tissues, mock-inoculated control plant tissues, plant tissues of zone 1, and plant tissue of zone 2 (columns; X variables), and the variables for relative transcript abundance of antioxidant genes, VB6 concentration, fungal biomass (rows; Y variables). Data were acquired from the analysis of relative transcript abundance, PN concentrations, and fungal biomass for potato sprouts*-R*. *solani* AG3 (10 antioxidant genes) [21 rows × 12 columns]. The data matrix for Arabidopsis-*R. solani* consisted of biological replicates of control fungal tissues, mock-inoculated control plant tissues, plant tissues with necrotic lesions (columns; X variables), and the variables for relative transcript abundance of antioxidant genes, VB6 concentrations, fungal biomass (rows; Y variables). Data were obtained from the analysis of 11 antioxidant genes (11 antioxidant genes) [15 rows × 13 columns].

The principal component analysis (PCA) was performed to determine which of the genes were most affected, and also to check if they were linearly correlated with VB6 concentration and fungal biomass in each interaction. Therefore, PCA loading coefficient plots (coupled with *p*-values) and loading biplots for the effect of VB6 concentration, fungal biomass, and the relative transcript abundance of genes in each tissue type were built for the visualization of correlations between the X and Y variables using the pc (corr) (i.e., correlation scaled loadings of the correlation between Y variables and X scores based on the variable importance of X as scores and loadings) setting in SIMCA-P+. Mean-centering and UV scaling were used for data normalization.

## Results

### Distinct Patterns of VB6 Gene Expression, Differential PN Concentration, and Fungal Biomass Among Potato Cultivars and Arabidopsis Lines

**FIGURE 2:**
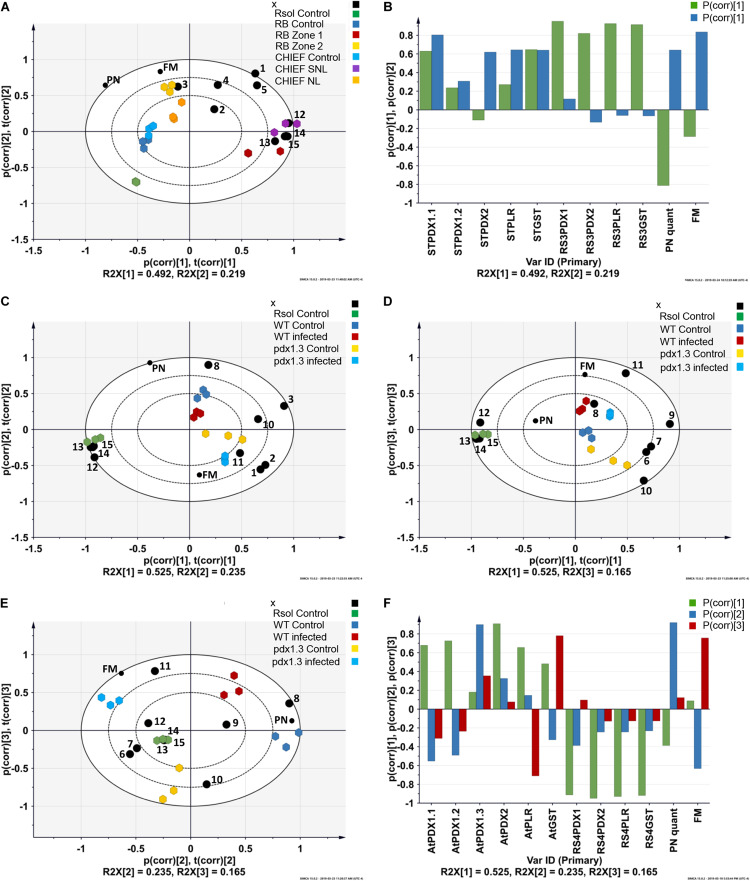
Principal component analysis (PCA) loading biplots for the effect of transcriptional abundance of antioxidant genes, PN concentrations, and fungal biomass on control, infected tissues in **(A)** potato sprouts-*R*. *solani* AG3 interaction, and **(C,D,E)** and Arabidopsis*-R*. *solani* AG4 interaction (*P* < 0.05). Principal component analysis (PCA) loading coefficient plots for the effect of transcriptional abundance of antioxidant genes, PN concentrations, and fungal biomass on control, infected tissues in **(B)** potato sprouts-*R*. *solani* AG3, and **(F)** Arabidopsis*-R*. *solani* AG4. The scaled loading vectors pc (corr) and t (corr) are displayed for the first and the second component for potato- *R*. *solani* AG3 interaction and the first, the second, and the third component for Arabidopsis*-R*. *solani* AG4 interaction. Outer ellipses represent the Hotelling’s T^2^ at a 95% confidence interval. R^2^X represents the fraction of the sum of squares of the two principal components. *ST*, *Solanum tuberosum*; *AT*, *Arabidopsis thaliana*; RS3, *R. solani* AG3; RS4, *R. solani* AG4. Black circle represents genes. 1 = *STPDX1*.*1*, 2 = *STPDX1*.*2*, 3 = *STPDX2*, 4 = *STPLR*, 5 = *STGST*, 6 = *ATPDX1*.*1*, 7 = *ATPDX1*.*2*, 8 = *ATPDX1*.*3*, 9 = *ATPDX2*, 10 = *ATPLR*, 11 = *ATGST*, 12 = *RsolPDX1*, 13 = *RsolPDX2*, 14 = *RsolPLR*, 15 = *RsolGST*. FM, fungal biomass; PN, vitamin B6 (pyridoxine) concentration; RB, Russet Burbank; CHIEF, Chieftain; Zone1, Tissue surrounding necrotic lesion; Zone 2, Necrotic lesion; Rsol, *R. solani* AG3 in the potato interaction and *R. solani* AG4 in the Arabidopsis interaction; WT, wild type.

To explore sources of variance within transcript levels, VB6 concentrations (measured as vitamer PN) and fungal load expressed as biomass, we used PCA. The analysis revealed tight groups with no outliers (*P* < 0.05) (Supplementary Figure S3). The principal components (PCs) which are the PCA axes are identified (Figures 2A,C,D,E). The amount of correlation between the expression levels of a gene, VB6 concentration and fungal biomass and a PC is designated by the extent of their loadings as indicated on the PC axes (Figure 2 and Supplementary Tables S1, S2). This approach identifies genes, VB6 concentration and fungal biomass having expression levels that are highly positively or negatively correlated with a given PC.

The strength of the correlations is pathosystem-dependent (Figure 2). In potato-*R. solani* AG3, PC1 and PC2 explained the majority of the variance in the dataset with tight grouping that led to the separation between the treatments (i.e., infected and control). Also, selected antioxidant genes were highly linked with the interaction zone as shown in the PCA-loading biplot of the potato-*R. solani* AG3 interaction (Figure 2A). In Arabidopsis –*R. solani* interaction, only PC2 and PC3 but not PC1 explained most of the variance in the dataset (Figures 2C–E). Interestingly, in both plant-fungus interactions, the PCA analysis showed that both fungal biomass and VB6 concentration contributed significantly to disease development (Figures 2B,F and Supplementary Tables S1, S2).

### Accumulation of ROS in *R*. *solani* Mycelia and Potato Plant Tissues Is Indicative of Oxidative Stress

**FIGURE 3:**
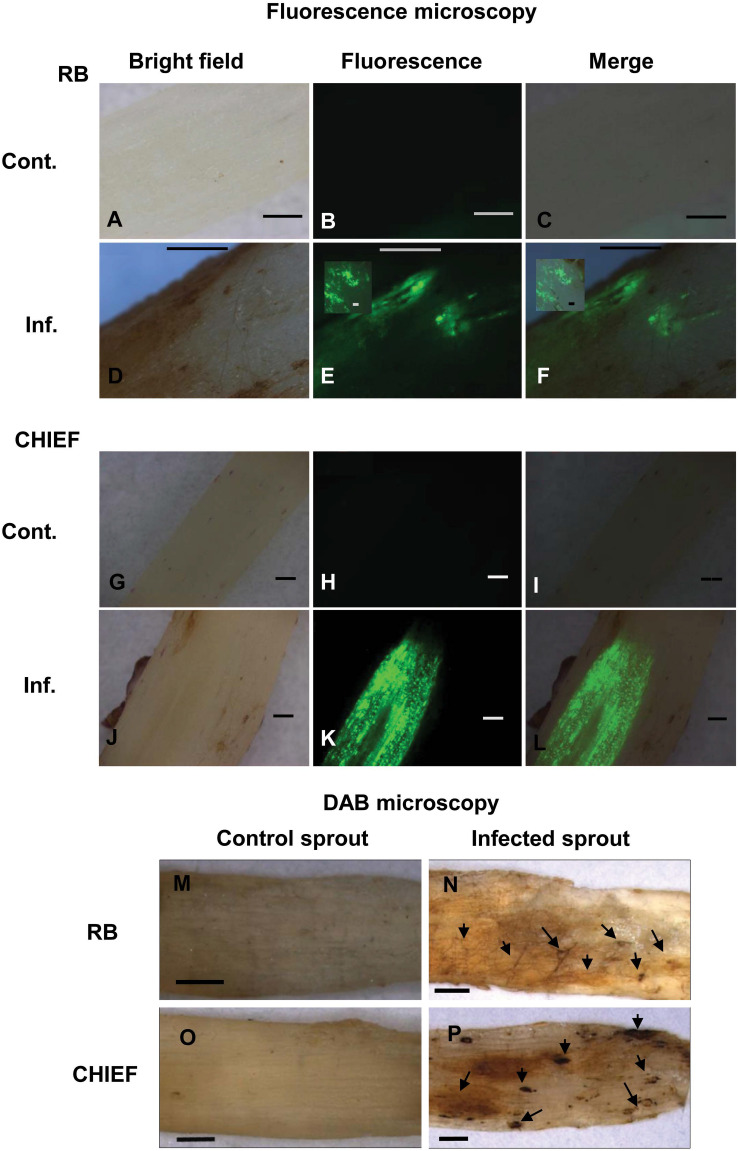
Intracellular production of ROS **(A–L)** and detection of H_2_O_2_ accumulation using DAB staining **(M–P)** during disease development in the potato-*R. solani* AG3 interaction. ROS production was visualized using H_2_DCF-DA in control and infected potato sprouts. Control *S*. *tuberosum* sprouts, with no evidence of endogenous ROS **(A,O)**. Infected tissues during disease development **(D–F,J–L)**. H_2_O_2_ accumulation was visualized by DAB staining in control and infected potato sprouts. Arrows indicate dark brown precipitate developed at necrotic and surrounding areas **(N,P)**. Control potato sprouts **(M,O)** with no evidence of endogenous H_2_O_2_ at 120 HPT. *R*. *solani* mycelia and *S*. *tuberosum* sprout **(N,P)** with H_2_O_2_ accumulation at 120 HPT. RB, Russet Burbank; CHIEF, Chieftain; Cont., control; Inf., infected. Bar = 1 mm.

Detection of ROS in the fungal hyphae and plant tissues was made possible by fluorescent H_2_DCF-DA and non-fluorescent DAB methods in Potato-*R. solani*. Tissues of mock-inoculated control of potato cv. Russet Burbank and Chieftain sprouts examined under bright field (Figures 3A,G), displayed no green fluorescence using the H_2_DCF-DA probe (Figures 3B,C,H,I). Under bright field (Figures 3D,J) infected potato cv. Russet Burbank and Chieftain sprouts with *R. solani* displayed intense green fluorescence (Figures 3E,F,K,L). The generation of ROS, primarily H_2_O_2_ and superoxide (O^–^_2_) in response to *R*. *solani* was assayed *in situ* using DAB staining. Tissues of infected potato cv. Russet Burbank sprouts displayed more brown precipitate pattern when compared to potato cv. Chieftain at necrotic and surrounding areas (Figures 3N,P, respectively). Digitally analyzed images of the same areas, using ImageJ software, resulted in higher disease score of 50.4% in Russet Burbank versus 32.4% in Chieftain. Control uninfected tissues did not show any browning (Figures 3M,O, respectively).

### Potato cv. Russet Burbank Had More Fungal Load Than Potato cv. Chieftain

**FIGURE 4:**
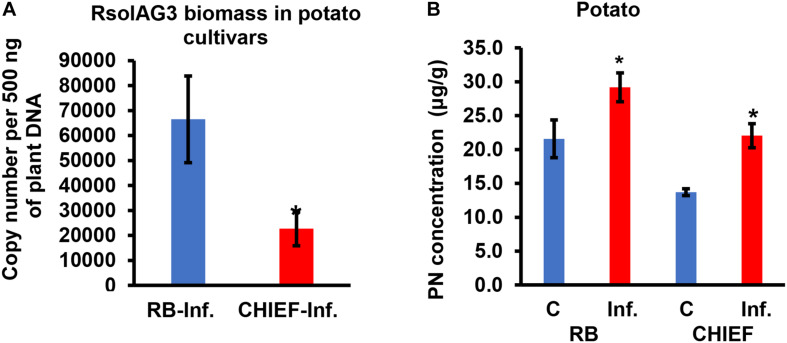
Quantification of fungal gDNA **(A)** and concentration (μg.g^–1^) of pyridoxine (PN) **(B)** in infected potato sprouts of two cultivars (RB and CHIEF). Fungal gDNA was measured using real-time quantitative PCR with 500 ng of plant samples. Asterisk indicates significant relative transcript abundance ratios between the control and the infected tissues using least significant difference (LSD) test (*P* < 0.05). RB, Russet Burbank; CHIEF, Chieftain; C, control; Inf., infected.

Analysis of the transcript for the *R. solani* G3PDH gene as an indicator of fungal load *in planta* confirmed that potato cv. Russet Burbank plants had more fungal biomass than the moderately tolerant potato cv. Chieftain. Potato cv. Russet Burbank displayed a statistically higher copy number of fungal gDNA compared to the moderately tolerant cv. Chieftain by 2.9-fold (Figure 4A), indicating that fungal biomass of the virulent strain 114 was closely correlated with stolon interaction, high loading coefficient values of fungal biomass were observed (Supplementary Tables S1, S2 and Figures 2B,F).

### Enhanced VB6 Content Has Only Limited Impact on Stress Performance in Potato Cultivars

VB6 analysis revealed strong recovery rates of 98.5 ± 1.5% for pyridoxine (PN). The method of detection limit was 0.124 μg.g^–1^ with a matrix effect of 102.41 ± 7.4%. Vitamin B6 (PN) content in plant tissues before and after inoculation with *R. solani* AG3 were linked with potato tissues of both cultivars based on loading coefficient values (Supplementary Table S1 and Figures 2A,E). Without pathogen inoculation, the constitutive levels of VB6 were significantly (*P* < 0.05) higher in the susceptible potato cv. Russet Burbank (1.6-fold) compared to the tolerant potato cv. Chieftain. After infection, VB6 content in infected potato tissues increased by 1.7-fold (*P* < 0.05) in potato cv. Chieftain, whereas in potato cv. Russet Burbank, the VB6 content slightly increased by1.4-fold change (Figure 4B).

### The Interaction Zone Shows Significant Shifts of Potato-Derived VB6 and GST Antioxidant Genes, Impacting Disease Resistance Against *R*. *solani*

**FIGURE 5:**
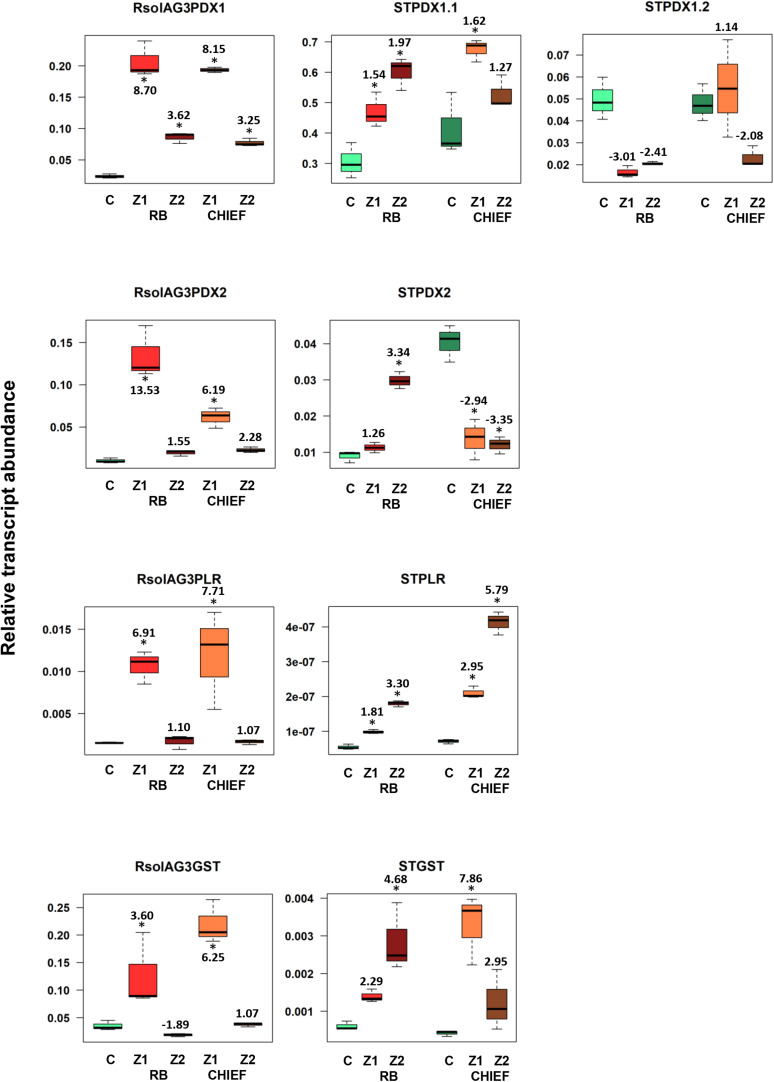
Relative transcript abundance of antioxidants encoding genes expressed in potato-*R. solani* AG3 interaction. Vitamin B6 (ST*PDX1*, ST*PDX2*, and ST*PLR*), and ST*GST* genes of the pathogen *R. solani* AG3 (*RsolAG3*) and the host, *Solanum tuberosum* (ST) in control and in infected and surrounding tissues at 120 HPI during *R. solani* AG3 and potato sprouts interaction. C: control; *R. solani* AG3 grown alone or potato sprouts inoculated with sterile with PDA. Zone1: tissue surrounding necrotic lesion. Zone 2, tissue of necrotic lesion; RB, Russet Burbank; CHIEF, Chieftain. Asterisk indicates significant relative transcript abundance ratios between the control and interaction using least significant difference (LSD) test (*P* < 0.05). Fold change is calculated in relation to the control at 120 HPI. *PDX1* and *PDX2*, *Pyridoxine biosynthesis* genes; *PLR*, *Pyridoxal Reductase*; *GST*, *Glutathione S-Transferase*.

The transcript abundance of potato antioxidant genes, *STGST* (p, 65%), and the VB6 *de novo STPDX1.1* (p, 63%) were positively correlated with potato cultivar and with the interaction zone sampled (i.e., zone 1 versus zone2). In the susceptible potato cv. Russet Burbank, a significant fold increase in relative transcript abundance of *STPDX1.1* (1.97-fold) and *STGST* (4.68-fold) was observed in zone 2. In the tolerant potato cv. Chieftain, an opposite trend was observed. The surrounding tissues in zone 1 significantly expressed fold increase of *STPDX1.1* (1.62-fold) and *STGST* (7.86-fold) (Figure 5). Gene expression of *STPDX2* was tightly linked to potato cultivar. A strong activation of *STPDX2* in tissues of zone 2 of potato cv. Russet Burbank with a 3.34-fold increase, and a downregulation in zone 1 and zone 2 in potato cv. Chieftain (−2.94 and −3.35-fold decrease, respectively). Despite the low loading value (p, 27%), the VB6 salvage pathways *STPLR* showed a significant upregulation of the relative transcript abundance in zones 1 and 2 in both potato cultivars, but with higher levels in potato cv. Chieftain (Figure 5). *STPDX1.2*, the non-catalytic homolog, showed a biologically significant down-regulation in both cultivars (up to a maximum of −3.01-fold decrease in cv. Russet Burbank) except in potato cv. Chieftain, the relative transcript abundance in zone 1 remained similar to the control tissues (1.14-fold) (Figure 5).

### Expression of Fungal–Derived VB6 Biosynthetic and *GST* Antioxidant Genes Are Not Correlated With Basal Resistance of Potato Cultivars but Are Tissue Zone-Driven

A notable upregulation of the fungal-derived genes encoding the vitamin B6 *de novo* and salvage pathway genes, and *GST* was observed during disease development of *R. solani* AG3 on sprouts of cv. Russet Burkank and Chieftain potato cultivars. Fungal genes with high loadings for PC1 were noted for *RsolAG3PDX1, RsolAG3PDX2, RsolAG3PLR*, and *RsolAG3GST* (Supplementary Table S1) and were abundantly expressed and tightly linked with interaction zone 1 (i.e., tissues of surrounding areas of necrotic lesions of both potato cultivars) (Figures 2A,B). Significant fold increase of transcript abundance of these genes ranged from a minimum of 3.60 for *RsolSG3GST* to a maximum of 13.53 for *RsolAG3PDX2* (Figure 5).

### Confirmation of Arabidopsis Null Mutants and Presence of ROS During the Interaction With *R. solani*

To explore the possibility of the role of VB6 in plant disease resistance, we have compared the expression of fungal-derived and plant-derived genes of Arabidopsis wild type Col-0 and *pdx1.3* mutants. Homozygous mutant plants were identified by genotyping with gene-specific primers flanking the insertion sites (Supplementary Figure S2). RT-PCR analysis revealed that no *ATPDX1.3* transcripts were detected in the *pdx1.3* mutant plants (Figure 8). These data indicate that the pdx1.3 are null mutants of the *ATPDX1.3* genes. These homozygous mutant lines were used for the further experiments as described below.

**FIGURE 6:**
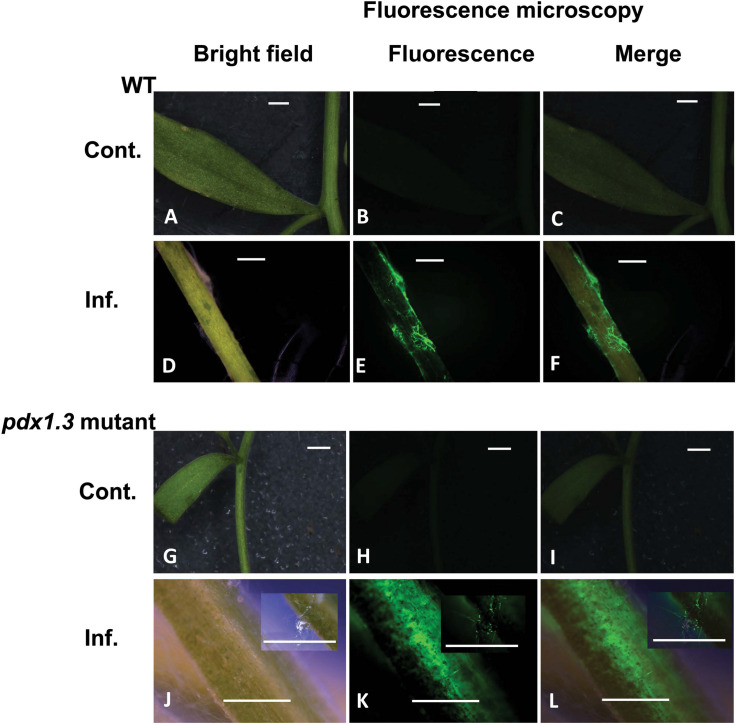
Intracellular production of ROS during disease development in the Arabidopsis-*R. solani* AG4 interaction. Control not infected tissues under bright field **(A)** and under fluorescence **(B,C)** show no ROS production. Infected tissues **(D)** under bright field and under fluorescence **(E,F)** with ROS production as visualized using H2DCF-DA. Control mutant line **(G)** under bright field and under fluorescence **(H,I)** show no ROS. Infected mutant line under bright field **(J)** showing disease development (inset) and under fluorescence (**K,L**, insets) showing ROS production as visualized using H2DCF-DA. RB, Russet Burbank; Cont., control; Inf., infected; Col-o or WT, wild type. Bar = 1 mm.

Tissues of mock-inoculated control of Arabidopsis wild type Col-0 and pdx1.3 mutant lower stems (Figures 6B,C,H,I) did not display any green fluorescence. An intense green fluorescence was detected in the tissues of Arabidopsis wild type and pdx1.3 mutant lower stems (Figures 6E,F,K,L), which supports the presence of oxidative stress during the interaction.

### The *pdx1.3* Arabidopsis Mutants Showed More Susceptibility to *R. solani* Coined With Lower Amounts of VB6

**FIGURE 7:**
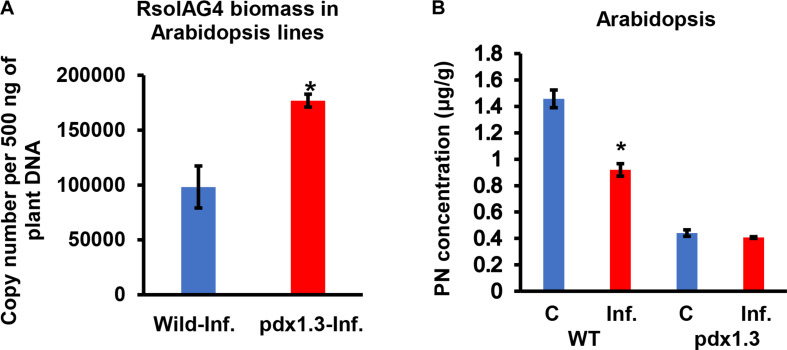
Quantification of fungal gDNA **(A)** and concentration (μg/g) of Pyridoxine (PN) **(B)** in tissues of two Arabidopsis lines (WT, wild type, pdx1.3 mutant) during disease development of *R. solani* AG4 on Arabidopsis, 72 h post-infection (HPI). Fungal gDNA was measured using real-time quantitative PCR with 500 ng of plant samples. Asterisk indicates significant difference between control and infected tissues at *P* < 0.05. WT, wild type; C, control; Inf., infected.

Arabidopsis *pdx1.3* plants were more susceptible to *R. solani* infections than the Arabidopsis wild type Col-0. The stems of the *pdx1.3* mutant Arabidopsis lines had significantly higher fungal load, expressed as gDNA copy number of A76 strain than that in the wild type plants by 1.80-fold (Figure 7A). Prior to infection, the constitutive VB6 content in Arabidopsis wild type was significantly higher (*P* < 0.05) by 3.31-fold compared to *pdx1.3* mutant line. After infection a drop in the VB6 levels in infected tissues of Arabidopsis wild type (1.6-fold decrease) was observed and no change occurred in the pdx1.3 mutant line (Figure 7B). Due to heavy infections and tissue maceration, digital analysis of infected tissues was not performed.

### Arabidopsis Vitamin B6 Genes and *ATGST* Mark a Potentially Adequate Defense Response During Disease Development

**FIGURE 8:**
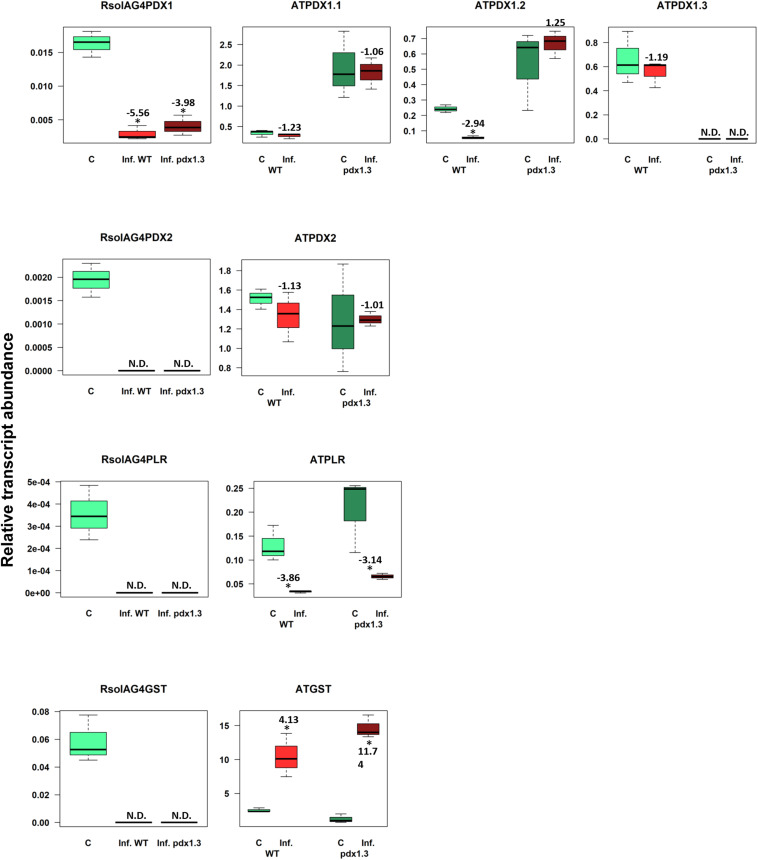
Relative transcript abundance of antioxidants in Arabidopsis*-R*. *solani* AG4 interaction. Vitamin B6 (*PDX1*, *PDX2*, and *PLR*), and *GST* genes of both the pathogen *R*. *solani* AG4 (*RsolAG4*) and the host, *Arabidopsis thaliana* (*AT*) in control and infected tissues at 72 HPI during *R*. *solani* AG4 and Arabidopsis lower stems interaction. C, control; *R*. *solani* AG4 grown alone or *Arabidopsis* lower stems inoculated with sterile PDA. Inf., infected; WT, wild type. Asterisk indicates significant relative transcript abundance ratios between the control and the interaction using least significant difference (LSD) test (*P* < 0.05). Fold change is calculated in relation to the control at 72 HPI. *PDX1* and *PDX2*, Pyridoxine biosynthesis genes; *PLR*, *Pyridoxal Reductase*; *GST*, *Glutathione S-Transferase.*

The VB6 biosynthesis gene *ATPDX1.3* is tightly linked with disease development based on coefficient loading values (Supplementary Table S2 and Figure 2F). After infection, levels of *ATPDX1.3* transcripts were similar to those in the control of the wild type. No transcripts were detected in the pdx1.3 mutant lines. As expected, higher transcript levels of *ATPDX1.1* (5.68 and 6.52 fold increases) and *ATPDX1.2* (2.19 and 11.89 fold increases) were found in both control and infected tissues of the pdx1.3 mutant lines compared to the wild type decreases (Figure 8). Interestingly, the relative transcript abundance for *ATPDX1.2* in the wild type infected tissues showed a significant 4.33-fold decrease in comparison to the control (Figure 8). The plant-derived VB6 salvage pathway gene *ATPLR* and the antioxidant *ATGST* gene had a significant role in Arabidopsis–*R. solani* AG4 interaction as estimated by the strong loading coefficient values (Supplementary Table S2). Expression profile patterns of *ATPLR* was down-regulated while expression of *ATGST* was induced in both the wild type and the pdx1.3 mutants. Fungal-derived vitamin B6 genes and *GST* were suppressed during the disease development of *R. solani* AG4 on Arabidopsis. Only *RsolAG4PDX1* was detected in the plant-infected tissues of both the wild type and the pdx1.3 mutant (Figure 8).

## Discussion

During disease development in plants, both the host and pathogen deploy a tight control and regulation of ROS production where the latter can function as a signaling factor leading to more fungal spread or suppression by the host defense machinery (Barna et al., 2012; Foley et al., 2016). This is reflected by unique differential expression of the antioxidant genes, including VB6 for different pathosystems. In this study, we compared the differential co-expression patterns of fungal and host VB6 and *GST* genes across the two potato cultivars.

This research further supports our previous findings (Samsatly et al., 2018) that both the pathogen and the host employ unique expression strategies in the necrotic lesion and in the surrounding tissue. The co-expression and accumulation of plant and pathogen vitamin B6 and other antioxidant genes in potato cultivars might be critical for ROS management and disease development and pathogenicity. To further study the factors governing the tight balance of ROS and the role of VB6, we investigated in this current work whether different potato cultivars containing variable amounts of vitamin B6 (Mooney et al., 2013) exhibit differential degrees of susceptibility to *R*. *solani* AG3. The experimental set-up described in this study provides further support to our previous findings (Samsatly et al., 2018) that both the pathogen and the host employ unique expression strategies of VB6 and *GST* antioxidant genes in the bordering tissue (zone 1) and in the necrotic tissue (zone 2). The use of multivariate analysis enabled us to draw meaningful biological associations of gene expression of VB6 biosynthetic pathways, *GST* antioxidant genes, VB6 content, and fungal biomass with potato genotypes exhibiting different degrees of susceptibility to *R. solani.* Our assumption that potato genotypes having different susceptibility background to *R. solani* would express unique VB6 and *GST* antioxidant genes profiles is established.

In the susceptible potato cv. Russett Burbank, the *de novo* VB6 biosynthetic genes *STPDX1.1*, *STPDX2*, *STGST*, and the VB6 salvage gene *STPLR* are upregulated in necrotic tissues (zone 2) in a manner consistent with increasing levels of VB6 mounting a pathogen response, but maintaining low expression levels in tissues bordering defense (zone 1). In sprouts of the tolerant potato cv. Chieftain infected with *R. solani*, an opposite transcript profile was established. A sharp increase in transcript abundance of these genes except for *STPDX2* in the bordering tissue (zone1) was detected with increasing levels of VB6 content. This fine-tuning expression strategy of VB6 and *GST* genes, is most likely that the tolerant potato plants must sustain ROS levels at the site of infection to defend against the pathogen and to stimulate the defense response, yet keep ROS levels lower in regions (zone 1) not affected by the pathogen to maintain tissue integrity and viability. In potato, all *STPDX* genes are expressed with the highest expression of *STPDX1.2* in sink areas such as tubers, roots, and stolons (Mooney et al., 2013). The role of the catalytic homolog *STPDX1.1* in response to biotic and biotic stress and its involvement during disease development is established (Moccand et al., 2014; Samsatly et al., 2018). It is noteworthy that the non-catalytic *STPDX1.2* is the most sensitive of *STPDX*s to biotic stress in this study.

We show that the expression of the *STPDX1.2* is strongly downregulated in sprouts under biotic stress in both infected potato genotypes compared to the controls (this study; Samsatly et al., 2018), whereas its catalytic homologs are not. Our results do not support the decisive regulatory role of *STPDX 1*.*2* with *STPDX 2* (Dell’Aglio et al., 2017) as established in certain crops (Zhang et al., 2014), but likely supports the negative regulatory role of STPDX 1.2 with *STPDX 2* in the tolerant potato genotype to *R. solani.* The differential regulation of VB6 could also involve cross-talk between *de novo* and the salvage gene pathway. This may imply that the salvage pathway may compensate for the absence of *STPDX2* with corresponding high expression levels of the *STPLR* in the tolerant potato cv. Chieftain. Probably, the requirement of the salvage pathway for disease resistance in potato is essential. At this point, we cannot fully account for the observed downregulation of *STPDX1.2* and *STPDX2*. An interesting finding that may be worth pursuing in the future is to understand how this diminution affects the regulation of VB6 biosynthesis.

In an attempt to understand how *R. solani* can regulate the levels of ROS for developmental and virulence purposes, we also compared the regulation of the fungal-derived VB6 genes of the biosynthetic pathways (*de novo* and salvage) and the antioxidant *GST* in cv. Russet Burbank and Chieftain potato cultivars. Contrary to their counterpart in the plant, the activation of all VB6 encoding genes and *GST* was similar and comparable between both cultivars indicating they are essential for fungal pathogenicity. It is noteworthy that the escalated expression of all the genes were detected exclusively in areas surrounding the necrotic lesions (zone 1). This corroborates previous results of another study on gene regulation of *R. solani* antioxidants such as *CAT, GST*, and VB6 (Samsatly et al., 2018) indicating that transcript abundance of these genes are tissue-specific. The results of tissue specificity mesh well with the limited data published on the VB6 expression in different infected tobacco tissues (Denslow et al., 2005). Upon infection of tobacco leaves with *P. syringae* pv. *Phaseolicola*, levels of tobacco *PDX1* expression were higher in the area surrounding the infiltration area. This increase most likely indicates that pathogens invest more on turning their antioxidant arsenal outside the killing zone in the necrotic lesion in order to expand further.

To provide direct evidence supporting the role of potato *PDX* genes in plant biotic defense against *R. solani*, we examined the phenotypes of Arabidopsis mutants with defect in the *de novo* VB6 biosynthesis pathway against infections by *R. solani* AG4. We compared the changes of VB6 biosynthetic gene, *ATPDX1.3* and VB6 (PN) contents in the wild type and *pdx1.3* plants before and after inoculation with *R. solani* AG4 strain A76. We selected Arabidopsis mutant defective in *PDX1.3* instead of *PDX1.2* because it is more requisite than *PDX1.1* for VB6 synthesis (Titiz et al., 2006; Tambasco-Studart et al., 2007), and has a role in disease resistance against *Pseudomonas syringae pv*. tomato in Arabidopsis compared to *ATPDX1.1* (Zhang et al., 2014, 2015). In this study, we examined the fungal load levels of *R. solani* and compared the *in-planta* fungal growth on the wild Col-0 and the mutant plants *pdx1.3*. Analysis of *R. solani* AG4 PDX1 gene, indicative of *R. solani* fungal growth *in planta*, confirmed that *pdx1.3* mutant plants had consistently high levels of fungal load expressed as fungal biomass than the wild type plants. Coined with this significant increase of *R. solani* gDNA copies in the *pdx1.3* mutant, was the reduction of the VB6 vitamer PN compared to the infected wild-type. These results indicate that mutations in *PDX1.3* gene resulted in increased susceptibility to *R. solani*. Similar trends were reported in studies dealing with interaction of the necrotrophic *Botrytis* with Arabidopsis *pdx1.3* mutant lines or tomato *slpdx1.3* silenced lines that led to increased disease severity (Zhang et al., 2014, 2015). As expected, silencing of *PDX1.3* but not *PDX1.2* resulted in a decreased level of VB6 in the mutant plant. This is in agreement with what has been previously published (Wagner et al., 2006; Tambasco-Studart et al., 2007). Recent studies support the antioxidant role of VB6 and that it can modulate plant defense by regulation of the antioxidant status in plants that are under biotic stress. Such regulation is distinctively expressed and depends on the crop, tissue infected and the type of the pathogen such as in the case of tobacco-*P. syringae* pv. *Phaseolicola*, tomato-*Botrytis cinerea*, and tomato-*Pseudomonas syringae* pv (pst) or Arabidopsis*-Pseudomonas syringae* pv-Arabidopsis (Zhang et al., 2014, 2015). Taken together, molecular and biochemical data signify that VB6 and its *de novo* biosynthetic pathway are essential for the regulation of defense response through the modulation of cellular antioxidant capacity.

In this study, we also evaluated the expression of VB6 *de novo* (*PDX2, PDX1.2, PDX 1.1*) and the salvage pathway genes (*ATPLR)* and *GST* antioxidant gene in the *pdx1.3* mutant line, and in the wild type before and after infection. Expression analysis of the VB6 *de novo* biosynthetic pathway genes was similar in the control and infected wild and mutant lines. It has been reported that *Arabidopsis* (*ATPDX1.2*) is a pseudoenzyme that acts as a positive regulator of vitamin B6 abundance during abiotic stress (Moccand et al., 2014). The drop of VB6 vitamer PN content is most likely due to the drastic down-regulation of *ATPDX1.2* in the infected wild type. On the other hand, the expression of *ATPLR*, a vital component in the VB6 salvage pathway, was down-regulated in both infected lines which can also explain the drop in the VB6 content. This might also mean less ability of the Arabidopsis lines to resist the pathogen given the role of *PLR* in detoxification of ROS during biotic and abiotic stress (Morissette et al., 2008; Chamoun and Jabaji, 2011; Gkarmiri et al., 2015; Samsatly et al., 2015). It is noteworthy that *ATGST* gene was substantially overexpressed in the wild and mutant lines of Arabidopsis during disease development as compared to the mock-inoculated controls of both lines. The high induction of *ATGST* is likely required to scavenge excess ROS in response to *R. solani* infection and bring the generated toxic oxygen metabolites to balanced level between production and quenching (Torres, 2010; Sharma et al., 2012; Das and Roychoudhury, 2014).

We also evaluated the expression *R. solani* VB6 biosynthetic *de novo* genes (*RsolPDX1, RsolPDX2*), the salvage gene (*RsolPLR*) and *RsolGST*. Interestingly, most of the fungal VB6 and *GST* genes were not detected and *RsolPDX1* was significantly suppressed. This pattern of down-regulation can result in an overflow of ROS in infected areas similar to what is found in other plant-necrotroph systems which appear to play an essential role during infection and initiation of the oxidative burst. For example, leaves of ivy pelargonium (*Pelargonium peltatum*) infected with *Botrytis cinerea*, exhibited a strong nitric oxide (NO) burst and hydrogen peroxide (H_2_O_2_) accumulation to arrest disease progression through NO-dependent reversible inhibition of catalase (Floryszak-Wieczorek et al., 2007).

PCA analysis identified vitamer PN as an important contributor during disease development in the potato-*R. solani* and Arabidopsis-*R. solani* interactions. Analysis of PN in potato genotypes before and after inoculation with *R. solani* by LC-MS showed that in the absence of the pathogen, the VB6 contents in potato cv. Russet Burbank and cv. Chieftain were approximately 21.6 and 13.7 μg.g^–1^, respectively, which are comparable to VB6 contents generally found in different potato genotypes (Mooney et al., 2013). On the other hand, the VB6 content in *pdx1.3* mutant line of Arabidopsis was reduced by 70% lower as compared to that of the wild-type plants. This observation is in agreement that *pdx1.3* plants grown in sterile culture medium contain reduced VB6 content (Zhang et al., 2014). After infection of potato genotypes by *R. solani* AG3, the content of PN in the susceptible cv. Russet Burbank and in the moderately tolerant cv. Chieftain significantly increased by 1.4 and 1.7-fold, respectively. The highest boost of PN in cv. Chieftain as compared to cv. Russet Burbank might be a factor leading to increased resistance to *R. solani*. This and the expression of VB6 *PDX1.1, STPLR, and GST* in tissues bordering the necrotic lesion is another factor that might determine the degree of susceptibility to *R. solani*. We put forward the assumption that the most important factor during disease development is not dependent on only how much PN is present prior to infection, but it is dependent on the ability of each plant to maintain or boost the production of PN at the right time and the right place.

After infection by *R. solani* AG4, the VB6 content in Arabidopsis wild-type was significantly reduced by 37%. However, the VB6 content in the infected *pdx1.3* mutant line was not affected and was similar to pdx1.3 mutant lines before infection. Our results indicate that reduced amounts of VB6 content lead to more Rhizoctonia infections in *pdx1.3* mutant lines. These results, along with the down-regulation of PDX1 in response to *R. solani* infections indicate that reduced biosynthesis and reduced VB6 content most likely represents a natural defense response in certain plants such as Arabidopsis, tomato, and tobacco against pathogenic infection (Denslow et al., 2005; Zhang et al., 2014, 2015). In contrast to what has been reported for Arabidopsis and other crops, it is not yet clear why the increased content of VB6 in potato genotypes led to more disease in the susceptible cultivar. Nevertheless, the data in this study provide direct evidence on the critical role of VB6 content in plant disease resistance.

It is common knowledge that ROS is generated at locations of attempted invasion through an oxidative burst during disease development (Alvarez et al., 1998; Lehtonen et al., 2008; Torres, 2010; Foley et al., 2016). ROS production was also reported in different anastomosis groups of *R*. *solani* that are pathogens of potato, soybean, sugar beet, and wheat. The induced ROS were also linked to the regulation of VB6 antioxidant genes and their collective possible role in oxidative stress alleviation (Taheri and Tarighi, 2011; Foley et al., 2016; Samsatly et al., 2018). Oxidative damage can be prevented in both the host and the pathogen when their antioxidant machinery is efficiently used (Apel and Hirt, 2004; Sharma et al., 2012). Microscopic analysis of *Rhizoctonai solani* AG3 and AG4 infections on stolons and stems confirmed that *Rhizoctonia* disease development in potato sprouts and Arabidopsis provokes an oxidative stress in fungal hyphae and plant tissues and could be related to the oxidative status of the fungus as well as the plant. Our data clearly showed that ROS formation was induced in the tissues of infected cv. Russet Burbank and Chieftain sprouts, and Arabidopsis wild type and *pdx1.3* mutant lower stems. Tissues of infected potato cv. Russet Burbank sprouts displayed stronger brown precipitate pattern when compared to Chieftain specifically in zones 1 and 2. This indicates that cv. Russet Burbank is experiencing a higher oxidative state and is less successful in quenching the H_2_O_2_ produced in the infected tissues.

## Conclusion

This work portrays the importance of VB6 during disease development of *R. solani* over the different potato cultivars or different lines of Arabidopsis. Our study showed a distinct pattern of expression, PN concentration, and fungal load expressed as biomass between the cv. Russet Burbank and Chieftain. The characterization of Arabidopsis *pdx1.3* mutant line challenged with *R. solani* offers convincing evidence into the role of VB6 in maintenance and enhancement of plant tolerance against *R. solani* AG4. The results obtained suggests the VB6 role in resistance to oxidative stress resulting from biotic interaction of *R. solani* with its natural hosts. Last, the regulation and production of VB6 is under tight control and is an essential determinant of disease development of *R. solani* with potato or Arabidopsis.

## Data Availability Statement

All datasets generated for this study are included in the article/Supplementary Material.

## Author Contributions

JS and SJ: conception and design of the study and interpretation of data for the work. JS and SB: acquisition of data for the study and analysis of data for the work. JS, SB, and SJ: manuscript revision and approval. All authors contributed to the article and approved the submitted version.

## Conflict of Interest

The authors declare that the research was conducted in the absence of any commercial or financial relationships that could be construed as a potential conflict of interest.

## Footnotes

1 http://imagej.nih.gov/ij/

2 http://signal.salk.edu/tdnaprimers.2.html

## Supplementary Material

FIGURE S1 Working conditions of Agilent 6540 Accurate-Mass QTOF.

FIGURE S2 Genotyping for homozygous pdx1.3 mutants (SALK086418) and wild (Col-0) Arabidopsis plants using SALK gene-specific primer pairs and T-DNA primer LB1.3 along with SALK086418-RP.

FIGURE S3 Principle component analysis score plots (PC1/PC2) for the effect of transcriptional abundance of antioxidant genes, PN concentrations, and fungal biomass on control, infected tissues in **(A)** pathosystem I (PC1/PC2), and **(B–D)** pathosystem II (PC1/PC2, PC1/PC3, and PC2/PC3) (*P* < 0.05). The ellipse represents the Hotelling T2 at a 95% confidence interval. Three biological replications were performed per treatment. Q2 (cum); cumulative fraction of the total variation of the X’s that can be predicted by the extracted components, R2X; the fraction of the sum of squares of the two principal components.

TABLE S1 *p*-values of principal component analysis loading coefficient plots for potato-*R. solani* AG3 interaction.

TABLE S2 *p-*values of principal component analysis loading coefficient plots for the Arabidopsis-*R. solani* AG4 interaction.
