# Supplementary figures and images for: Vitamin B6 Is Under a Tight Balance During Disease Development by Rhizoctonia solani on Different Cultivars of Potato and on Arabidopsis thaliana Mutants
Source: Front Plant Sci. 2020 Jun 24;11:875. doi: 10.3389/fpls.2020.00875 (PMC7327096; doi:10.3389/fpls.2020.00875)

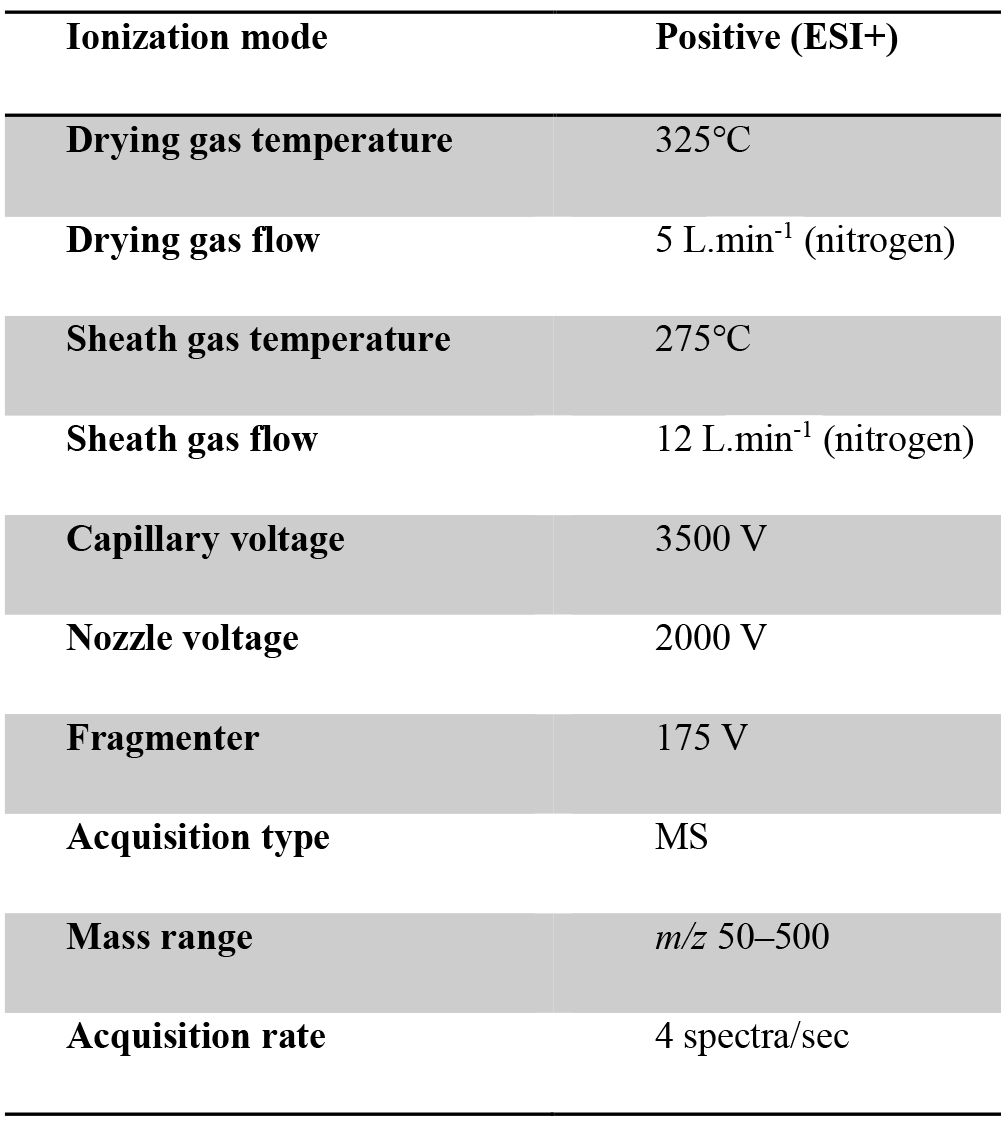

Supplement: FIGURE S1 — Working conditions of Agilent 6540 Accurate-Mass QTOF. [file Image_1.TIF]

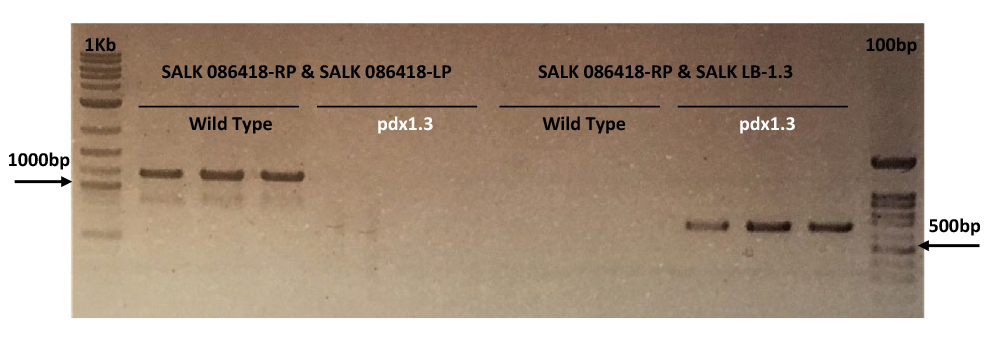

Supplement: FIGURE S2 — Genotyping for homozygous pdx1.3 mutants (SALK086418) and wild (Col-0) Arabidopsis plants using SALK gene-specific primer pairs and T-DNA primer LB1.3 along with SALK086418-RP. [file Image_2.TIF]

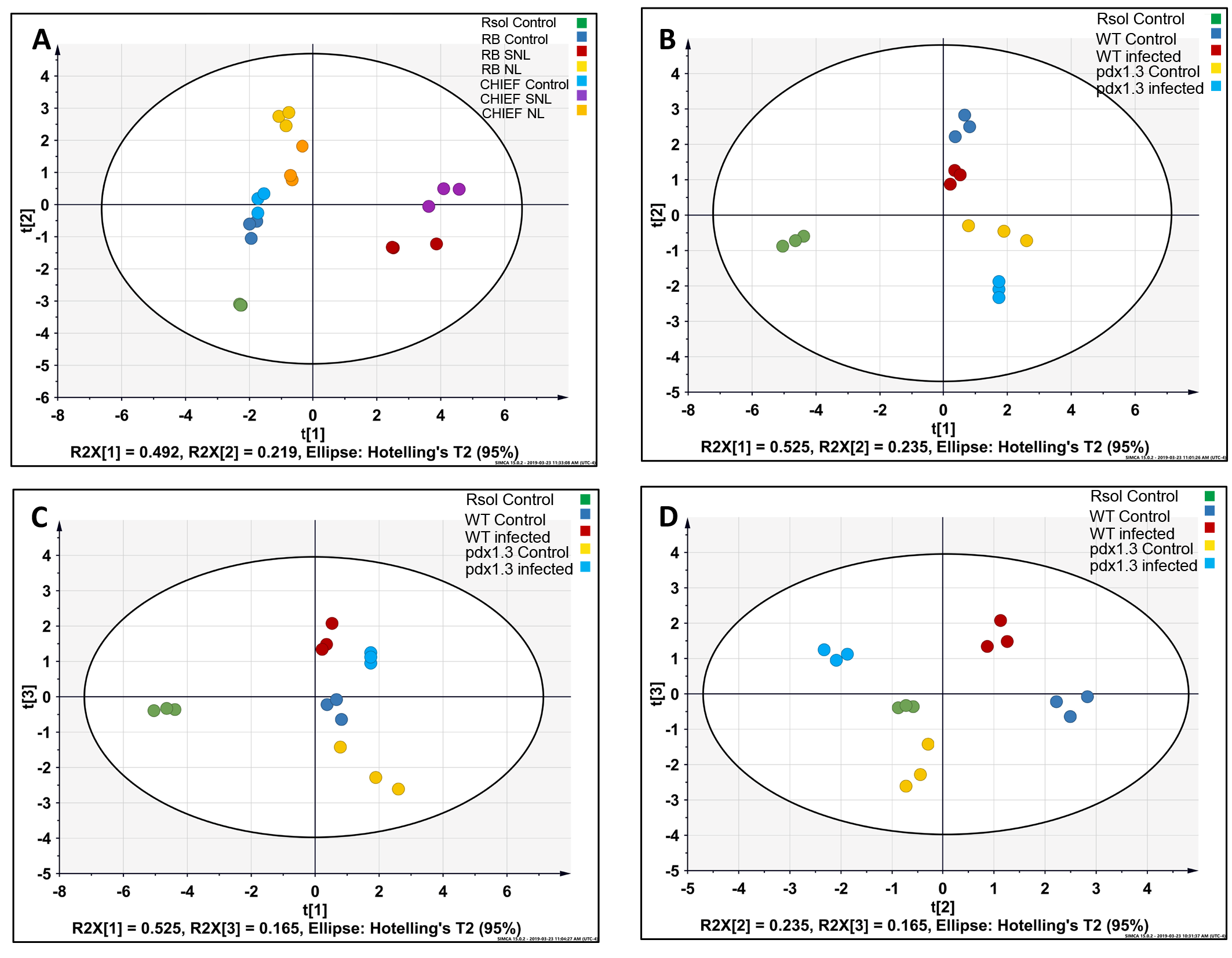

Supplement: FIGURE S3 — Principle component analysis score plots (PC1/PC2) for the effect of transcriptional abundance of antioxidant genes, PN concentrations, and fungal biomass on control, infected tissues in (A) pathosystem I (PC1/PC2), and (B–D) pathosystem II (PC1/PC2, PC1/PC3, and PC2/PC3) (P < 0.05). The ellipse represents the Hotelling T2 at a 95% confidence interval. Three biological replications were performed per treatment. Q2 (cum); cumulative fraction of the total variation of the X’s that can be predicted by the extracted components, R2X; the fraction of the sum of squares of the two principal components. [file Image_3.TIF]
