# Supplementary material for: Vitamin B6 Is Under a Tight Balance During Disease Development by Rhizoctonia solani on Different Cultivars of Potato and on Arabidopsis thaliana Mutants
Source: Front Plant Sci. 2020 Jun 24;11:875. doi: 10.3389/fpls.2020.00875 (PMC7327096; doi:10.3389/fpls.2020.00875)
Supplement: TABLE S1 — p-values of principal component analysis loading coefficient plots for potato-R. solani AG3 interaction. [file Table_1.DOCX]

**Supplementary Table 1.** *p* values of principal component analysis loading coefficient plots for potato-*R. solani* AG3 interaction.

| **Variables** | **p(corr)[1]** | **p(corr)[2]** |
| --- | --- | --- |
| *STPDX1*.*1* | 0.63 | 0.80 |
| *STPDX1*.2 | 0.24 | 0.31 |
| *STPDX2* | -0.11 | 0.62 |
| *STPLR* | 0.27 | 0.64 |
| *STGST* | 0.65 | 0.64 |
| *RsolPDX1* | 0.95 | 0.12 |
| *RsolPDX2* | 0.82 | -0.14 |
| *RsolPLR* | 0.93 | -0.06 |
| *RsolGST* | -0.81 | 0.64 |
| PN concentration | -0.81 | 0.64 |
| Fungal biomass | -0.29 | 0.84 |

**Supplementary Table 2.** *p* values of principal component analysis loading coefficient plots for the Arabidopsis-*R. solani* AG4 interaction.

| **Variables** | **p(corr)[1]** | **p(corr)[2]** | **p(corr)[2]** |
| --- | --- | --- | --- |
| *ATPDX1*.*1* | 0.70 | -0.55 | -0.31 |
| *ATPDX1*.2 | 0.73 | -0.49 | -0.24 |
| *ATPDX1*.3 | 0.18 | 0.90 | 0.36 |
| *ATPDX2* | 0.91 | 0.33 | 0.078 |
| *ATPLR* | 0.66 | 0.15 | -0.71 |
| *ATGST* | 0.48 | -0.33 | 0.78 |
| *RsolPDX1* | -091 | -0.39 | 0.10 |
| *RsolPDX2* | -0.95 | -0.25 | -0.13 |
| *RsolPLR* | -0.93 | -0.24 | -0.13 |
| *RsolGST* | -0.92 | -0.23 | -0.13 |
| PN concentration | -0.39 | 0.92 | 0.12 |
| Fungal biomass | 0.09 | -0.63 | 0.76 |
